# Editorial: “Recent Advances in Gamma/Delta T Cell Biology: New Ligands, New Functions, and New Translational Perspectives”

**DOI:** 10.3389/fimmu.2015.00371

**Published:** 2015-07-21

**Authors:** Dieter Kabelitz, Julie Déchanet-Merville

**Affiliations:** ^1^Institute of Immunology, University of Kiel, Kiel, Germany; ^2^CNRS UMR 5164, Université de Bordeaux, Bordeaux, France

**Keywords:** γδ T cells, infection, tumor immunity, phosphoantigen, IL-17

Since their discovery in the mid-1980s, interest in the immunological significance of γδ T cells has been subject to oscillations. The initial excitement over the unexpected discovery of a second T cell receptor (TCR) was followed by years of uncertainty as to the biological importance of these ambivalent T cells. Major breakthroughs led to the identification of specific and unique antigens for the γδ TCR and accumulating evidence now shows that γδ T cells play a major role in local immunosurveillance, thereby controlling tumorigenesis. Since 2004, biannual international γδ T cell conferences are held to bring together experts in basic and clinical γδ T cell research. To make accessible and synthesize the body of knowledge that has been put together, to date, we have organized a “Research Topic” on γδ T cells consisting of a collection of original articles and focused reviews written by leading experts in the field. The idea of this Research Focus was to present the current status and “hot topics” as well as clinical perspectives on γδ T cell research.

## γδ T Cells: Differentiation, Activation, and Signaling

The signaling pathways governing γδ T cell differentiation and activation have been discussed in contributions from Carl Wares’s (1) and Bruno Silva-Santos’s (2) groups. Ribeiro et al. discuss the control of γδ T cell activation and differentiation by distinct classes of cell surface receptors, namely (i) the TCR, (ii) costimulatory receptors (with a focus on CD27), (iii) cytokine receptors, (iv) NK receptors, and (v) inhibitory receptors. They further summarize how activation of γδ T cells can be controlled by the TCR as well as by activating NK receptors. To terminate γδ T cell responses, several inhibitory receptors can deliver negative signals, notably PD-1 and B- and T-lymphocyte attenuator (BTLA) (2). Bekiaris et al. focus on the cytokine control of innate γδ T cells, and discuss the role of IL-7 in being critical for thymic development of γδ T cells by regulating the survival of progenitor cells and inducing V(D)J recombination within the TCRγ gene locus. IL-7 also supports homeostatic proliferation of γδ T cells and regulates surface expression of BTLA in a STAT5-dependent manner (1).

In recent years, IL-17 has been identified as an essential cytokine that regulates the recruitment of neutrophils during an inflammatory response. IL-17 must be rapidly available in an acute infection. αβ T cells producing IL-17 (Th17 cells) require specific antigenic stimulation and an appropriate cytokine milieu for differentiation. γδ T cells have been identified as an important “innate” source of rapid initial IL-17 production, which is thought to occur without specific TCR triggering (3). Several years ago, the group of Immo Prinz generated a unique mouse model to monitor early steps of γδ T cell development (4). Using these reporter mice, they demonstrated that IL-17 producing γδ T cells develop during the embryonic period and persist in adult mice as self-renewing, long-lived cells (5). Interestingly, Wei and colleagues have provided evidence that the TCR repertoire of such naturally occurring IL-17-producing murine γδ T cells is highly restricted, with little or no junctional diversity, regardless of their anatomical origin. These findings strongly suggest that antigen recognition is involved in the establishment and/or function of such “innate” IL-17 producing γδ T cells (6). Further aspects of IL-17 producing γδ T cells, also addressing their role in various pathophysiological conditions, are discussed in the review article by Patil et al. (7).

In addition to production of cytokines, such as IL-17, γδ T cells can induce maturation of dendritic cells (DCs) and B cells. Petrasca and Doherty report on the upregulation of the expression of CD86, HLA-DR, IL-6, and TNFα in both DC and B cells upon coculture with human Vγ9Vδ2 T cells, whereas other cytokines, such as IFNγ or IL-4, were differentially induced in DC versus B cells (8). Collectively, their data indicate that γδ T cells can drive the expression of antigen presenting cell (APC)-associated markers in both DC and B cells (8). Interaction with neighboring cells is a key feature of γδ T cells. This is particularly true for tissue-resident γδ T cells. Witherden et al. have reviewed the multiple molecular interactions that have been characterized between skin-resident dendritic epidermal γδ T cells and keratinocytes. Among their many effector functions, epidermal γδ T cells are involved in wound repair, maintenance of epithelial homeostasis, and protection from malignant transformation (9).

A final aspect of γδ T cell differentiation and plasticity is presented in the report of Ziegler et al. (10). These authors made the surprising observation that the small population of human peripheral blood Vδ1 γδ T cells that simultaneously express CD4, can differentiate into *bona fide* αβ T cells in a process called transdifferentiation. The authors took all measures to avoid the potential contribution of possible artifacts that may influence their results. Their characterization of human CD4^+^Vδ1 γδ T cells as a source of extrathymic T cell development challenges current dogmas and opens interesting avenues for future research (10).

## Recognition of Pyrophosphate Antigens by Human γδ T Cells

A particularly exciting new area of γδ T cell research is the role of butyrophilin and butyrophilin-like molecules (which consist of all members of the B7 family of co-stimulators) in γδ T cell activation. A member of this family, Skint-1, has been shown as critical for positive selection of mouse Vγ5Vδ5 dendritic epidermal T cells (11). In humans, Butyrophilin 3A1 (BTN3A1) was recently found to play a critical role in the activation of Vγ9Vδ2 T cells by pyrophosphate molecules – collectively termed as “phosphoantigens” (pAgs). These intermediates of the eukaryotic mevalonate and the prokaryotic non-mevalonate pathway for isoprenoid synthesis have been identified as specific ligands for the Vγ9Vδ2 TCR that activate this subset of T cells at pico- to nanomolar (microbial pAgs) or micromolar (eukaryotic pAgs) concentrations. The recognition of metabolites that are produced by microbes [e.g., (*E*)-4-hydroxy-3-methyl-but-2-enyl pyrophosphate (HMBPP)] or that are overproduced by transformed eukaryotic cells [e.g., isopentenyl pyrophosphate (IPP)] provides a formal basis for the role of Vγ9Vδ2 T cells in both anti-infective and anti-tumor immunity (12). Interestingly, the recognition of non-peptidic small molecules is not restricted to human γδ T cells. As an example, Zeng et al. recently reported that small molecules, such as haptens, are recognized by specific murine γδ TCRs (13).

For many years, it has been enigmatic how pAgs are actually recognized by the human γδ TCR, and whether any “presenting” molecules are involved. A major breakthrough was the identification of an agonistic monoclonal antibody (called 20.1) that specifically triggers Vγ9Vδ2 T cell activation among peripheral blood mononuclear cells in a manner that is very similar to pAgs. This antibody is directed against CD277, a member of the B7 superfamily of butyrophilin (BTN) molecules. Detailed studies by Harly et al. identified an indispensable role of the BTN3A1 isoform in the activation of Vγ9Vδ2 T cells by pAgs (14). BTN3A1 is a transmembrane molecule with two Ig-like extracellular domains and an intracellular B30.2 domain. This discovery of the essential function of BTN3A1 in Vγ9Vδ2 T cell activation stimulated further research, particularly addressing the question whether BTN3A1 is directly involved in “presenting” pAgs to the γδ TCR.

In continuation of the above studies, Gennaro de Libero’s group has come up with new evidence that suggests the Ig-like extracellular domain of BTN3A1 can directly bind pAgs and that there is specific and direct interaction of soluble Vγ9Vδ2 TCR with the BTN3A1–pAg complex (15). Quite surprisingly, however, such a “presenting” function of the extracellular BTN3A1 domain could not be verified by Erin Adam’s group. Instead, Adam and colleagues corroborated there being a crucial role of the intracellular B30.2 domain, which may actually directly interact with pAgs (16). In their model, the intracellular B30.2 domain might sense increased levels of intracellular pAgs (due to infection or cellular transformation), possibly inducing a conformational change in the BTN3A1 molecule, which could then be recognized by the Vγ9Vδ2 TCR (16). However, the issue appears to be even more complicated. Using CHO cells reconstituted with chromosome 6 as the only source of human genes, Riaño et al. demonstrate a potential role of other genes on chromosome 6 in addition to BTN3A1 that work to activate human Vγ9Vδ2 T cells (17). The various proponents of the seemingly conflicting models on the capacity of extra- versus intracellular BTN3A1 domains to directly interact with pAgs provide a balanced overview on this controversial issue in this special Research Topic (18–20).

Beyond this, “the intracellular pAg sensing model” recently received additional support from John Trowsdale’s group. These authors also reported direct binding of HMBPP to the intracellular B30.2 domain (21). Furthermore, using a yeast-two hybrid screen, theses authors determined that the cytoskeletal protein plakin interacts with the cytoplasmic tail of BTN3A1 in a region proximal to the B30.2 domain. Such protein–protein interactions might be crucial in transmitting signals (e.g., conformational alterations of BTN3A1) from the γδ TCR upon pAg binding to the intracellular B30.2 domain (21). It is thus evident that many of the mechanistic details of BTN3A1 are still unclear, beyond the undisputed fact that it is indispensable for γδ T cell activation by pAgs.

Intriguingly, the mouse lacks BTN3A1 genes and homologous γδ TCR that would be able to recognize similar pAgs. Karunakaran and Herrmann (22) have studied the ontogenetic evolution of Vγ9, Vδ2, and *BTN3* genes. Their detailed database analysis suggests that these three genes have co-evolved in placental mammals, while they are lost in rodents and lagomorphs (22). Taken together, the controversial issue of the ménage à trois of Vγ9Vδ2 TCR, pAgs, and BTN3A1 awaits additional elucidation. We are grateful that the major players in this particular field have shared their results and thoughtful reflections for this Research Topic.

## γδ T Cells in Tumor Immunity

γδ T cells can kill a broad range of tumors of epithelial origin as well as many leukemias and lymphomas, and are further able to produce high levels of the anti-tumor cytokine, IFNγ. Given their HLA non-restricted method of ligand recognition, the role of γδ T cells in anti-tumor immunity has stimulated great interest to explore their potential for cancer immunotherapy (23). Interestingly, the production of endogenous pAgs, such as IPP, can be pharmacologically manipulated by nitrogen-containing bisphosphonates (N-BP), which inhibit the downstream processing of IPP, leading to increased levels of endogenous IPP that is sensed by Vγ9Vδ2 T cells (24). Based on this knowledge, intravenous application of the N-BP zoledronate together with low-dose IL-2 has been evaluated as a means of *in vivo* activation of γδ T cells in cancer patients (25). A recent survey has reviewed the available published studies on the *in vivo* activation and adoptive transfer of γδ T cells in cancer patients (26). Most likely, strategies aiming to activate γδ T cells *in vivo* will have to be combined with other treatment regimens to obtain optimal anti-tumor activity (27).

In addition to IPP and related pAgs, which serve as antigens for tumor-reactive Vγ9Vδ2 T cells, several cell surface-expressed antigens have been identified for tumor-reactive human non-Vδ2 γδ T cells. One example illustrating the versatility of γδ T cells is the endothelial protein C receptor (EPCR), which is an HLA-related molecule. We (Déchanet-Merville group) identified EPCR as a target for non-Vδ2 γδ T cells that is expressed on endothelial cells infected by cytomegalovirus (CMV) and is also similarly expressed on epithelial tumor cells (28). It is thus conceivable that non-Vδ2 γδ T cells have a similar potential for cancer immunotherapy as pAg-reactive Vδ2 T cells (23, 29). The recent discovery that CD1d, the closest structural homolog of EPCR, can present self lipids to the human Vδ1 TCR, also deserves further investigations in the domain of γδ T cell-mediated anti-tumor protection and function (30, 31). It should be borne in mind that the anti-tumor efficacy of γδ T cell subsets (Vγ9Vδ2, non-Vδ2) may, of course, vary between different tumor entities (32).

One of the basic observations supporting a role of immune surveillance in tumor development and progression is the sticking correlation between the proportion of tumor-infiltrating CD3^+^ T cells (TIL) and tumor progression and patient survival (33). γδ T cells can represent a significant proportion of CD3^+^ TIL in different tumors (34, 35). Hidalgo et al. analyzed the presence of γδ T cells among TIL in different types of breast carcinoma. Their results suggest a correlation between higher numbers of γδ TIL and a better prognosis in medullary breast carcinoma as compared to invasive ductal carcinomas, two subtypes of the Her2- and hormone receptor-negative (“triple-negative”) breast carcinoma (35).

It is clear, however, that an increased presence of γδ T cells within a tumor *per se* is not necessarily associated with a beneficial effect. As discussed by Lo Presti et al., there are multiple interactions of tumor-infiltrating γδ T cells within the local tumor microenvironment that strongly influence the functional outcome (36). Relevant factors include (but are not restricted to) tumor-derived immunosuppressive cytokines, such as TGF-β, locally expressed inhibitory molecules, such as PD-1, and myeloid-derived suppressor cells (MDSCs) located within the tumor stroma (36). Moreover, tumor-infiltrating γδ T cells themselves may exert suppressive activity (37) or promote the accumulation of MDSCs (38). Pro-tumoral activity of γδ T cells has also been demonstrated in several mouse models, and this is mainly mediated through their production of IL-17, which, in these instances, results in the attraction of immunosuppressive myeloid cells and promotion of angiogenesis. Depending on the priming signals in the tumor microenvironment, in addition to other γδ T cell subtypes, even pAg-reactive Vγ9Vδ2 T cells may acquire suppressive activity (39). In the context of tumor immunity, multiple scenarios may thus convert γδ T cells into suppressive cells, which can have counter-productive consequences for tumor immunity (40). Taken together, γδ T cells can exert both anti- and pro-tumorigenic activities, and it is a major challenge for future studies to determine how to specifically boost the anti-tumor effects of γδ T cells while simultaneously shunting their suppressive activity (41). This functional plasticity of mouse and human γδ T cells in the anti-tumor immune response has been extensively discussed by Lafont et al. (42).

To enhance the anti-tumor activity of γδ T cells, several strategies are under consideration. These include the use of antibodies to trigger Fc receptor-dependent ADCC, or the use of bispecific antibody constructs to cross-link the γδ TCR with tumor cell surface antigens. Seidel et al. have explored Fc-optimized anti-CD19 antibodies as well as CD19/CD16 bispecific antibodies to enhance γδ T cell-mediated killing of CD19^+^ B cell malignancies (43). For this purpose, they employed a label-free real-time cytotoxicity assay, which allows monitoring the tumor cell–γδ T cell interactions over prolonged periods of time (43). This system was also used by us (Kabelitz group) to demonstrate the efficacy of a Her2-Vγ9 “tribody” construct in enhancing lysis of Her2-expressing pancreatic adenocarcinoma cells by Vγ9Vδ2 effector T cells (34). A different approach to explore the potential use of γδ T cells for cancer immunotherapy would be to develop γδ T cell-based cancer vaccines. This strategy is based on the seminal discovery of Bernhard Moser’s group that found activated human Vγ9Vδ2 T cells may actually serve as “professional” APCs that pick up and process exogenous antigens and load them onto both MHC class II and MHC class I presentation pathways for recognition by CD4^+^ and CD8^+^αβ T cells, respectively (44). Toward this end, the capacity of activated human γδ T cells to take up tumor-derived antigens and to present processed peptides to tumor antigen-specific CD8^+^αβ T cells has been demonstrated (45). As discussed by Khan et al., the ease with which Vγ9Vδ2 T cells are expanded into large numbers *in vitro* offers an innovative strategy for the development of γδ T cell-based tumor vaccines (46).

For the adoptive transfer of *in vitro* expanded γδ T cells, a multitude of experimental protocols have been developed. One important aspect to bear in mind for this procedure is to design means to fully polarize γδ T cells toward an efficient anti-tumor functionality. Promotion of their IFNγ expression instead of IL-17 will certainly be an important step toward this aim (41). Along this line, Deniger and coworkers have summarized various strategies of *in vitro* expansion and further discuss additional perspectives involving genetic approaches to increase the efficacy of γδ T cell-based immunotherapy (47). A crucial point in this context is the efficient monitoring of γδ T cell subpopulations in the blood of cancer patients. We (Kabelitz group) have established a system to accurately determine absolute numbers of circulating Vδ1 and Vδ2 γδ T cells from a small sample of whole blood. This method enabled us to determine a threshold number of Vδ2 T cells per microliter blood below which no cytotoxic activity toward pancreatic adenocarcinoma tumor targets could be triggered by pAg or Her2-Vγ9 tribody (48). Another important step for the application of γδ T cell-based immunotherapy involves identifying the antigenic ligands recognized by γδ T cells on tumor cells as they may potentially be used as agonistic drugs either *ex vivo* for γδ T cell expansion or *in vivo* for active vaccination trials. Together, the collection of papers published in this Research Topic discuss critical issues pivotal for understanding the precise role of γδ T cells in tumor immunosurveillance or tumor immune evasion as well as highlighting the future potential of γδ T cell-based immunotherapies.

## γδ T Cells in Infection and Other Diseases

In view of the strong activation of human Vγ9Vδ2 T cells by microbial pAgs (notably HMBPP), it is not surprising to see significant expansion of peripheral blood γδ T cells during the acute phase of many infections (49). However, not all microbes produce such γδ T cell-stimulating molecules. The differential production of γδ T cell ligands by microbes can be used for diagnostic purposes to identify the presence of (mostly Gram-negative) bacteria and thus allow for a pathogen-specific immune fingerprinting. This inventive diagnostic application of γδ T cell research in the context of infectious diseases is highlighted in the Opinion Article by Eberl et al. (50).

While Vγ9Vδ2 T cells increase in numbers in many bacterial and parasitic infections, these cells are characteristically reduced in HIV infected individuals. Even upon efficient anti-retroviral therapy and CD4 T cell reconstitution, Vγ9Vδ2 T cell numbers usually remain low. Pauza and colleagues discuss the potential consequences of persistent low Vγ9Vδ2 T cell numbers in HIV infected individuals and argue in favor of designing innovative clinical trials to reconstitute normal levels of Vγ9Vδ2 T cells (51). Another example of viral infection with high relevance for γδ T cells is CMV. Our (Déchanet-Merville group) early studies have uncovered a remarkable expansion of Vδ2-negative γδ T cells in CMV-positive but not -negative kidney transplant recipients (52). Further studies have revealed multiple possible functions of CMV-induced γδ T cells, including direct anti-viral activity. Concomitant studies in mouse models point to a protective role of murine γδ T cells against CMV infection (53, 54). All these aspects including the cross-reactivity of CMV-reactive non-Vδ2 γδ T cells with certain epithelial tumor entities are discussed in a review article by Couzi et al. (55).

This Research Topic also addresses the role of γδ T cells in non-infectious diseases. In addition to infection and cancer, autoimmune diseases, and wound healing and burn injuries have been addressed by Latha et al. (56). This review summarizes the extensive evidence showing the involvement of γδ T cells in many pathophysiological conditions. An interesting specific example is reported by Marcu-Malina and coworkers (57) who observed a TNF-dependent induction of procoagulant tissue factor-1 (TF-1) in monocytes by zoledronate-stimulated γδ T cells. This seemed to occur specifically in γδ T cells from patients with systemic sclerosis (57). Last but not least, this Research Topic additionally covers the role of γδ T cells in the diagnosis of immunodeficiencies. TCR immunodeficiencies can affect both αβ and γδ T cells. While αβ T cells have been extensively studied, γδ T cells are frequently ignored, partly due to their numerical scarcity in circulation. Garcillán et al. discuss these issues in detail and present a useful diagnostic flowchart (58).

## Concluding Remarks

After 30 years of γδ T cell research, it is clear that these cells are intimately involved in the control of tissue homeostasis, infection, and malignancy. The identification of specific ligands for the γδ TCR provides strong support for the idea that γδ T cells are non-redundant to αβ T cells. Apart from the detailed knowledge of their physiological and pathophysiological significance, we are currently experiencing new exciting developments aimed at bringing γδ T cells into clinical medicine.

## Conflict of Interest Statement

The authors declare that the research was conducted in the absence of any commercial or financial relationships that could be construed as a potential conflict of interest.
